# PEG treatment is unsuitable to study root related traits as it alters root anatomy in barley (*Hordeum vulgare* L.)

**DOI:** 10.1186/s12870-024-05529-z

**Published:** 2024-09-13

**Authors:** Veronic Töpfer, Michael Melzer, Rod J. Snowdon, Andreas Stahl, Andrea Matros, Gwendolin Wehner

**Affiliations:** 1 Institute for Resistance Research and Stress Tolerance, Julius Kuehn-Institute (JKI) – Federal Research Centre for Cultivated Plants, Quedlinburg, Germany; 2 Department of Structural Cell Biology, Leibniz Institute of Plant Genetics and Crop Plant Research (IPK), Gatersleben, Germany; 3 Department of Plant Breeding, IFZ Research Centre for Biosystems, Land Use and Nutrition, Justus Liebig University, Giessen, Germany

**Keywords:** Barley, Drought stress, Genotypes, Polyethylene glycol, Root system

## Abstract

**Background:**

The frequency and severity of abiotic stress events, especially drought, are increasing due to climate change. The plant root is the most important organ for water uptake and the first to be affected by water limitation. It is therefore becoming increasingly important to include root traits in studies on drought stress tolerance. However, phenotyping under field conditions remains a challenging task. In this study, plants were grown in a hydroponic system with polyethylene glycol as an osmotic stressor and in sand pots to examine the root system of eleven spring barley genotypes. The root anatomy of two genotypes with different response to drought was investigated microscopically.

**Results:**

Root diameter increased significantly (*p* < 0.05) under polyethylene glycol treatment by 54% but decreased significantly (*p* < 0.05) by 12% under drought stress in sand pots. Polyethylene glycol treatment increased root tip diameter (51%) and reduced diameter of the elongation zone (14%) compared to the control. Under drought stress, shoot mass of plants grown in sand pots showed a higher correlation (*r* = 0.30) with the shoot mass under field condition than polyethylene glycol treated plants (*r* = -0.22).

**Conclusion:**

These results indicate that barley roots take up polyethylene glycol by the root tip and polyethylene glycol prevents further water uptake. Polyethylene glycol-triggered osmotic stress is therefore unsuitable for investigating root morphology traits in barley. Root architecture of roots grown in sand pots is more comparable to roots grown under field conditions.

**Supplementary Information:**

The online version contains supplementary material available at 10.1186/s12870-024-05529-z.

## Introduction

Climate change is one of the most prominent problems in the world [1, 2], whereby extreme weather events such as drought stress are expected to appear even more often in future with devastating negative consequences on the crop yield [3]. Barley (*Hordeum vulgare* L.), an important cereal crop for nutrition, brewing and feeding, has a higher drought tolerance than other cereals like wheat [4]. Barley is often used as model crop [5, 6] because it is easy to cultivate, and the genome has been available a long time ago [7]. Drought stress affects barley roots at first because roots are the main plant organ for water uptake. Increased root length and root to shoot ratio have been found to be desirable traits related to a higher drought tolerance [8]. However, investigation of roots under field conditions is complex and the root losses during sampling can be considerable, especially losses of fine roots [9]. Instead, simulated drought stress experiments in hydroponic systems using polyethylene glycol (PEG) as stressor have become increasingly common and are often used as the method of choice in molecular biology and screening experiments [10–12]. Advantages of hydroponic experiments with PEG-induced stress are the easy stress induction and the reproducibility, as previously shown for barley seedlings [13]. Furthermore, other sugars utilized for drought stress induction like mannitol are well known to be taken up by the roots and tissues [14]. The mechanism of PEG-triggered osmotic stress is to disrupt root water uptake [15] by increasing the osmotic pressure similarly to a reduced water availability in the soil causing drought stress [16]. However, the advantages of the system are partly offset by the number of disadvantages, i.e. a highly artificial system with non-representative root expansion in a fluid compared to soil compartments, induction of drought stress by disruption of water uptake instead of water shortage, to name only the most prominent. Other experiments related to drought stress and root growth rely on sand-based substrates to mirror more realistic soil scenarios [17] allowing to preserve most of the roots at harvest.

Root architecture adaptions to drought stress are diversely characterized in cereals [18]. In general, drought stress under field conditions leads to an increased root-shoot ratio and tap root growth [19, 20] as well as a reduced root dry weight, volume, diameter and lateral root formation [21, 22]. However, root length is generally increased under drought stress for tolerant genotypes, while sensitive ones showed a decrease in root length [22]. Another important root trait is the root density. It was reported that a high root hair density with longer root hairs can be beneficial for drought stress tolerance [23, 24]. Other advantageous characteristics to better cope with drought stress are a higher number of xylem vessels for enhanced water transport, a steeper root angle, a higher number of side roots, and higher total volume for physical strength [25]. Recently, different root ideotypes for improved drought stress tolerance of crops were described by Shoaib et al. [26]. The first ideotype, ideal for deep soil water availability, is characterized by a reduced root number on the soil surface layers and a higher root number in deep soil layers with increased root hair numbers and xylem diameter. The second ideotype, ideal for low homogenous water availability, is characterized by deep roots with long lateral roots, more root cortical aerenchyma, and a narrow root angle. A third ideotype, called ‘shallow roots’, is described with more surface roots at a wide root angle, dense fine roots, higher root hair numbers, and few deep roots to capture the water from the low rainfall events before it is lost by evaporation [27]. However, a universal ideotype for drought in a broader range of crops might not be applicable because roots of each crop interact and respond differently regarding the soil type and dicot crops might differently respond compared to monocot cereals. Thus, further studies are required for a better understanding of barley root adaptation and genotypic differences under drought stress.

The specific objective of the present study was to investigate barley root growth under drought stress in a hydroponic system using PEG as osmotic stressor compared to sand pot experiments. To investigate impacts on the whole plant, correlations between the plant shoot mass under these controlled conditions and the shoot mass under field conditions were made to determine which method, PEG or sand, leads to results that are more comparable to field trials under drought stress. In addition, a microscopic analysis was performed to check for the PEG uptake in the roots and to reveal possible unintended changes in root cell structure and architecture. Our hypothesis is that the osmotic stress related increase of root diameter is caused by PEG uptake. In contrast, drought stress exposure in sand pots reduces the root diameter. The response of plants to drought stress in sand pots may be more comparable to the response under field conditions than the response to drought stress in a hydroponic system. Overall, we expected changes in root architecture and cell structure exposed to the hydroponic PEG system causing overlapping effects on plant performance and thus are not representative for drought studies under real conditions.

## Materials and methods

### Plant material and experimental setup

Eleven diverse spring barley genotypes (Supplementary Table 1) from the IPK-SB224 panel [28] were provided by the gene bank of the Leibniz Institute of Plant Genetics and Crop Plant Research in Gatersleben (https://www.ipk-gatersleben.de/en/infrastructure/gene-bank/gene-bank-gatersleben), and were tested under hydroponic condition and in pots with sand. Hydroponic and sand pot experiments were performed four times each within five replications per genotype and treatment in a randomized complete block design. For both experiments, seedlings were germinated on watered filter paper in petri dishes for four days in darkness at room temperature (24 °C). Germinated seeds for the hydroponic experiments were transferred to neoprene rings into 20 l boxes containing 1 l Hoagland solution according to Shavrukov et al. [29] (Supplementary Fig. 1). Each box contained 30 plants fixed in neoprene rings (Ø 5 cm). For the pot experiments, germinated seeds were transferred to pots filled with 300 g sand. All pots were watered three times per week with 20 ml Hoagland solution until seven days after sowing (DAS). All plants grew with 10 h daylight at a temperature of 18 °C. Drought stress treatment started at 12 DAS for both experiments (Supplementary Fig. 1). Drought stress treatment under hydroponic condition was performed with 12.5% (w/v) PEG 6000. Plants under controlled condition were grown in Hoagland solution without PEG under the same conditions.

For the pot experiments, the control plants were watered three times a week with 25 ml Hoagland solution, while drought stressed plants received 10 ml three times a week from seven DAS onwards (Supplementary Fig. 1). Five plants were grown per treatment and genotype. Both experiments were performed in three independent replications after each other and were finished at 33 DAS (Supplementary Fig. 1). Afterwards, all plants were harvested, and leaves were cut and weighed to measure the shoot fresh mass. Then, leaves were dried at 105 °C for two days and weighed again for shoot dry mass. Roots were first carefully washed and then weighed for root fresh mass for both growth conditions. The weighted roots were stored in 15 ml falcon tubes with 50% ethanol (w/v). Roots were transferred to a transparent dish filled with water, in which root branches were carefully pulled apart for scanning and accurate data processing. The dish with the water and root was scanned using an Epson Expression 12000XL scanner (Seiko Epson K.K., Japan) and scan images were immediately analyzed by the WinRhizo software (Regent Instruments, Canada) at 34 DAS. After the root analysis, roots were dried at 105 °C for two days and weighed for root dry mass determination.

### Microscopic analysis

For microscopic analysis, roots of two genotypes (8 and 11) with a different response to drought stress (data unpublished) were collected at 33 DAS from one hydroponic experiment and prepared for histological and ultrastructural analysis using light- and transmission electron microscopy as described in Muszynska et al. [30]. ImageJ, an open-source software for image processing (https://imagej.net/software/fiji), was used to analyze tissue diameter of the root tip and the elongation zone of six primary and secondary roots per genotype and treatment.

### Field trials

Field experiments were conducted in 2021 and 2022 in Groß Lüsewitz, Germany (54°07’N, 12°33’E), a location characterized by a diluvial and almost sandy-loamy soil and a mean rainfall of 689 mm (https://www.julius-kuehn.de/vf/gross-luesewitz/*).* Ten spring barley genotypes (genotype 1–10) were grown under irrigated and drought stress conditions in rainout-shelter facilities with three replications per genotype and treatment. Plot size was 2.3 m² for 32 plots per irrigation treatment and border plots (cv Alexis in 2021, cv Amidala in 2022) in a randomized complete block design. Drought stress was applied at BBCH 13 for six weeks with a soil water capacity of 20% and the irrigated control with 50%. PlantCare Mini-Loggers (PlantCare Ltd., Sennhof 13, CH-8332 Russikon, Switzerland) were used to monitor soil water capacity (Supplementary Fig. 2). Shoot mass was harvested 133 DAS and weighed without ears and roots. Plant protection treatments were performed against powdery mildew with 0.25 l/ha of cyflufenamide (Vegas, Certis Europe B.V., Hamburg, Germany) at 71 DAS, against leaf rust with 3 l/ha epoxiconazole, pyraclostrobine and fluxapyroxade (Ceriax, BASF SE Limburger Hof, Germany) and against aphids with the insecticide agent lambda-cyhalothrine (Kaiso Sorbie, Nufarm GmbH & Co KG, Linz, Austria) at 99 DAS.

### Statistical analysis

The root-shoot ratio was calculated as the ratio of root mass and shoot mass (root mass/shoot mass). Dry matter content was calculated with the following equation:

1 $${\text{Dry matter content }}\left[ \% \right]{\text{ }} = {\text{ }}\left( {{\text{shoot dry mass}}/{\text{shoot fresh mass}}} \right){\text{ }} \times {\text{ }}100$$

Adjusted means, ANOVA (analysis of variance) and Fisher’s least significant difference (LSD) test (*p* < 0.05) were calculated for all traits measured in the hydroponic experiments using the following linear mixed model:

2 $${P_{ijkl}} = {{\mathbf{\mu}}} + {{\mathbf{g}}_i} + {{\mathbf{t}}_j} + {({\mathbf{gt}})_{ij}} + {\text{ }}Ex{p_k} + {\text{ }}{\left( {Exp:Rep} \right)_{kl}} + {\text{ }}{e_{ijkl}}$$

where P_ijkl_ is the observed phenotype of the *i*th genotype, in the *j*th treatment, the *k*th experiment (Exp) and the *l*th replication (Rep). µ is the general mean of the experiment, g_i_ is the *i*th fixed effect of the genotype, and t_j_ is the *j*th fixed effect of the treatment. (gt)_ij_ is the fixed factor of the genotype-treatment interaction. Exp_k_ is the *k*th random effect of the experiment and (Exp: Rep)_kl_ is the random factor of the *l*th replication in the *k*th experiment. e_ijkl_ is the error term. The fixed effects are indicated by bold lower-case letters.

The adjusted means, ANOVA and Fisher’s least significant difference test (*p* < 0.05) for the pot experiment were calculated with the equation:

3 $$\begin{aligned}{P_{ijklmn}} =& \mu + {{\mathbf{g}}_i} + {{\mathbf{t}}_j} +\\& {({\mathbf{gt}})_{ij}} + {\text{ }}Ex{p_k} + {\text{ }}{\left( {Exp:Rep} \right)_{kl}} + \\&{\text{ }}{\left( {Exp:Row} \right)_{km}} + {\text{ }}{\left( {Exp:Col} \right)_{kn}} + {\text{ }}{e_{ijklmn}}\end{aligned}$$

where P_ijklmn_ is the observed phenotype of the the *i*th genotype, the *j*th treatment, the *k*th Exp, *l*th Rep, the the *m*th Row and the *n*th column (Col). µ is the general mean of the experiment, g_i_ is the *i*th fixed effect of the genotype, and t_j_ is the *j*th fixed effect of the treatment. (gt)_ij_ is the fixed factor of the genotype-treatment interaction. Exp_k_ is the *k*th random effect of the experiment, (Exp: Row)_km_ is the random effect of the *m*th row in the *k*th experiment, (Exp: Col)_kn_ is the random effect of the *n*th column in the *k*th experiment. (Exp: Rep)_kl_ is the random factor of the *l*th replication in the *k*th experiment and e_ijklmn_ is the error term. The fixed effects are indicated by bold lower-case letters.

Adjusted means of shoot mass under field conditions were calculated with the equation:

4 $${P_{ijm}} = {{\mathbf{\mu}}} + {{\mathbf{g}}_i} + {{\mathbf{t}}_j} + {({\mathbf{gt}})_{ij}} + {\text{ }}Yea{r_m} + {\text{ }}{e_{ijm}}$$

where P_ijm_ is the observed phenotype of the the *i*th genotype, the *j*th treatment, the *n*th Rep, and the *m*th year, µ is the general mean of the experiment, g_i_ is the *i*th fixed effect of the genotype, and t*j* is the *j*th fixed effect of the treatment. (gt)_ij_ is the fixed factor of the *i*th genotype within the *j*th treatment. Year is the *m*th random effect of the year. e_ijm_ is the error term. The fixed effects are indicated by bold lower-case letters.

Data analysis was performed using the statistical programming language and environment R (version 4.1.1; R Core Team, 2021). Pearson coefficient of correlation was calculated based on adjusted means of genotypes investigated under field conditions (*n* = 10) and plots were created through the R package ‘corrplot’ [31]. Conduction of ANOVA, LSD and calculation of adjusted means were done by the R-packages ‘as.reml’ [32] and ‘lme4’ [33, 34]. The calculation of the significance levels between the genotypes of the root traits and between control and PEG treatment of root tip and elongation zone diameter were calculated by Students *t*- test.

## Results

### Root morphology

Eleven root related traits and three above ground parameters were investigated in a hydroponic system and for plants growing in pots filled with sand (Supplementary Table 2). Under hydroponic condition, significant (*p* < 0.05) genotype dependent reactions were observed (Table 1). Moreover, all traits showed a significant difference (*p* < 0.001) between control and PEG treatment (Table 1). While root-shoot ratio (dry and fresh), dry matter content and root diameter were increased by PEG treatment in all genotypes, all other traits were significantly decreased by the treatment (Supplementary Table 3).

**Table 1:** Analysis of variance (ANOVA) according to Eq. 2 for the investigated traits in the hydroponic system

Trait	Unit	Genotype	Treatment	GxT	LSD
Dry matter content	%	< 0.001	< 0.001	< 0.05	6.83
Shoot dry mass	g	< 0.05	< 0.001	< 0.001	0.11
Shoot fresh mass	g	< 0.001	< 0.001	< 0.01	0.52
Root dry mass	g	< 0.001	< 0.001	ns	0.01
Root fresh mass	g	< 0.001	< 0.001	ns	0.13
Root crossings	-	< 0.001	< 0.001	< 0.001	134.63
Root diameter	mm	< 0.001	< 0.001	ns	0.02
Root forks	-	< 0.001	< 0.001	< 0.001	684.94
Root length	cm	< 0.001	< 0.001	< 0.001	76.99
Root-shoot ratio dry	-	< 0.001	< 0.001	ns	0.04
Root-shoot ratio fresh	-	< 0.001	< 0.001	< 0.01	0.14
Root surface area	cm²	< 0.001	< 0.001	< 0.01	7.86
Root tips	-	< 0.001	< 0.001	< 0.01	327.73
Root volume	cm³	< 0.001	< 0.001	ns	0.07

*GxT* genotype and treatment interaction, *LSD* least significant difference, *ns* non-significant

For the sand pot trials, the parameter root dry mass showed no significant genotype effect and root-shoot ratio of fresh tissue was not affected by the stress treatment (Table 2). In contrast, all other traits showed significant (*p* < 0.001) genotype and treatment effects for the sand pot trials (Table 2). Interestingly, the root diameter under stress was significantly (*p* < 0.001) decreased by 14% (Supplementary Table 4). All other parameters responded as in the hydroponic system, while a lower increase for the fresh root-shoot ratio and dry matter content by PEG treatment in all genotypes was detected (Supplementary Table 4).

**Table 2:** Analysis of variance (ANOVA) according to Eq. 3 for the investigated traits of the sand pot trials

Trait	Unit	Genotype	Treatment	GxT	LSD
Dry matter content	%	< 0.001	< 0.001	< 0.05	1.88
Shoot dry mass	g	< 0.001	< 0.001	< 0.05	0.01
Shoot fresh mass	g	< 0.001	< 0.001	< 0.01	0.16
Root dry mass	g	ns	< 0.001	ns	0.01
Root fresh mass	g	< 0.01	< 0.001	< 0.05	0.17
Root crossings	-	< 0.001	< 0.001	< 0.01	45.39
Root diameter	mm	< 0.001	< 0.001	< 0.05	0.04
Root forks	-	< 0.001	< 0.001	< 0.01	422.74
Root length	cm	< 0.001	< 0.001	ns	46.72
Root-shoot ratio dry	-	< 0.001	< 0.001	< 0.05	0.08
Root-shoot ratio fresh	-	< 0.001	ns	ns	0.21
Root surface area	cm²	< 0.01	< 0.001	< 0.05	7.42
Root tips	-	< 0.001	< 0.001	< 0.001	120.96
Root volume	cm³	< 0.01	< 0.001	< 0.01	0.11

*GxT* genotype and treatment interaction, *LSD* least significant difference, *ns* non-significant

**Fig. 1:**
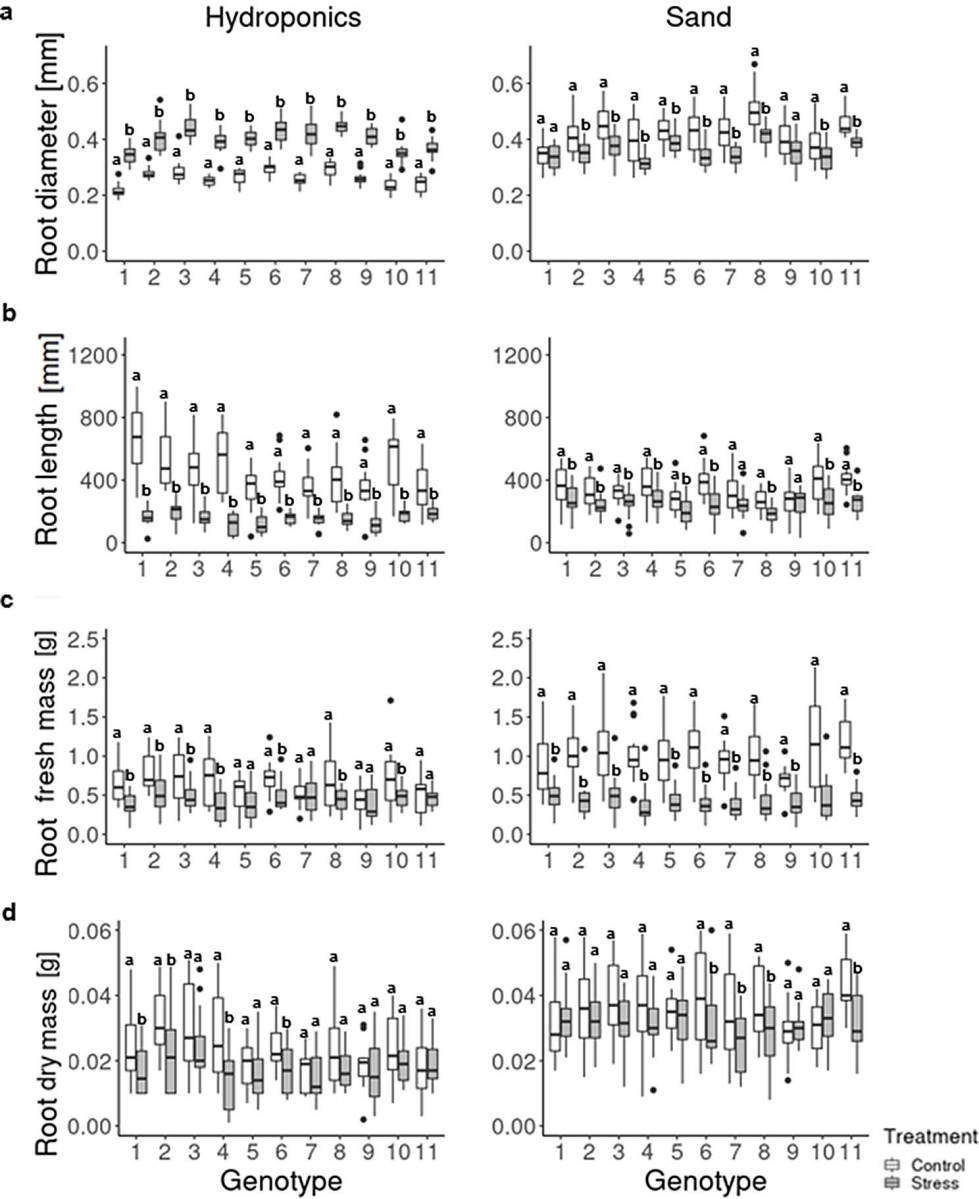
Comparison of the most important root traits between the two growth conditions of eleven genotypes of spring barley. **a** Root diameter, **b** Root length, **c** Root fresh mass and **d** Root dry mass of eleven genotypes and treatment effect, *n* = 15. Significance (*p* < 0.05) between treatments was tested with Student’s *t*-test

Furthermore, the reaction of the roots to the drought stress treatment was analyzed for each genotype. The most interesting observation was a significantly increased root diameter under hydroponic condition for all genotypes (Fig. 1a), whereas ten of eleven genotypes showed a significant decrease (*p* < 0.05) in root diameter under the stress treatment in pots with sand. Genotype 1 showed no significant reaction for the root diameter under the stress treatment in sand. The root length decreased under stress treatment for both growth conditions (Fig. 1b). However, the control roots in the hydroponic system were longer than the control roots in the sand pots.

All genotypes differed in their response to the root fresh mass under hydroponic condition, while genotypes 5, 7, 9 and 11 showed no alterations after PEG treatment (Fig. 1c). However, the PEG treatment led to a decrease in the root fresh mass in the other genotypes and the ones grown in sand pots (Fig. 1c). The root dry mass was only significantly negative affected for genotypes 1, 2, 4 and 5 under hydroponic condition and genotypes 6, 7, 8 and 11 for the sand pot trials (Fig. 1d). The decrease in these traits was different under the two growth conditions with higher root dry mass in all genotypes and treatments in the plants grown in sand pots (Fig. 1d). Overall, roots grown in the hydroponic system with PEG as stressor developed differently from the root that grew in pots filled with sand.

### Root anatomy and PEG uptake

**Fig. 2:**
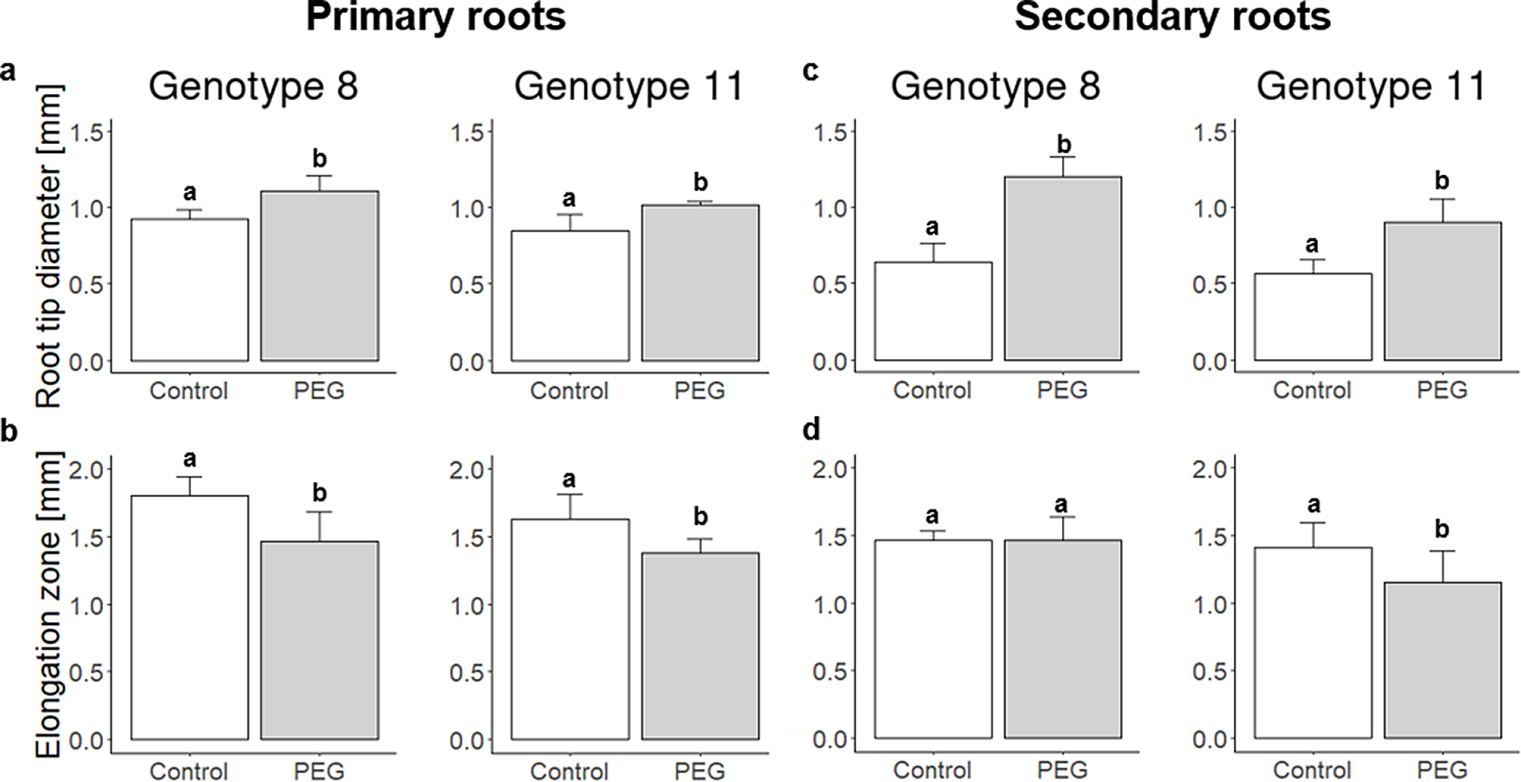
Diameter of the root tip and elongation zone of the primary (**a**,** b**) and secondary roots (**c**,** d**) in genotypes 8 (Morex) and 11 (BCC436). Image analysis of histological transversal sections of genotypes 8 and 11 analyzed by ImageJ. Significance (*p* < 0.05) between treatments was tested with Student’s *t*-test

Based on these results and the fact that the root diameter significantly increases through a PEG treatment, the hydroponic grown roots were examined microscopically to determine the potential cause of the root thickening. Two different genotypes (8 and 11) were used for the microscopy. First, the root tip diameter and the diameter of the elongation zone were measured for the two investigated genotypes. As expected (Fig. 1a), the root tip diameter was significantly (*p* < 0.05) increased by PEG in primary and secondary roots (Fig. 2a, c). Interestingly, the diameter of the elongation zone was significantly (*p* < 0.05) decreased by PEG except for genotype 8 in secondary roots (Fig. 2b, d).

**Fig. 3:**
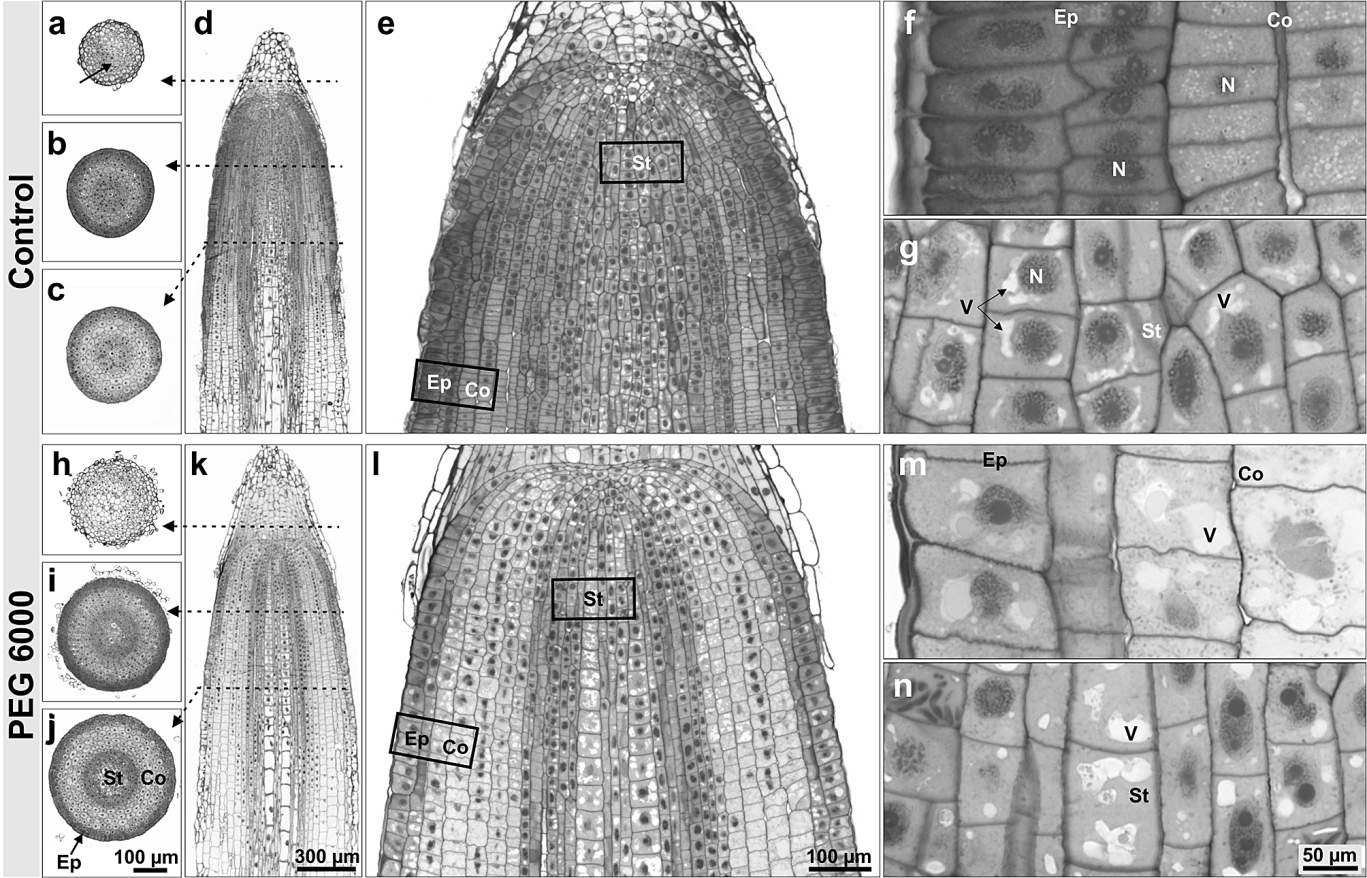
Light microscopy analysis of barley root tips of *Hordeum vulgare* cv. Morex. Transversal (**a-c**, **h-j**) and longitudinal histological sections (**d-g**, **k-n**) of root tips of plants grown under control condition (**a-g**) and drought stress treatment with 12.5% (w/v) PEG 6000 (**h-n**). Detailed micrographs of stele cells (**g**,** n**), epidermis and cortex cells (**f**,** m**). *Co* cortex; *Ep* epidermis, *N* nucleus; *St* stele, *V* vacuole

Second, light microscopy was used to investigate the root anatomy. Here, it is clearly recognizable that the PEG-treated roots had a wider root diameter than the control roots (Fig. 3a-c, h-j). Furthermore, the cross-section images of PEG treated roots showed swollen root cells independent on the root layer (Fig. 3h-n) compared to the control roots, which are more compact (Fig. 3a-f). The PEG treated root cells contained enlarged vacuoles across all root layers (Fig. 3m-n), which were not present in the cells of the control roots (Fig. 3f-g). The transmission electron microscopy images emphasized this phenomenon (Supplementary Fig. 3). In addition, the cytoplasm was generally not dense with PEG and contained less proteins and ribosomes compared to the control (Supplementary Fig. 3). In some cases (cortex), the cytoplasm was very loose and organelles such as the nucleus, ER, mitochondria or plastids were clearly altered and many components were dissolved by PEG (Fig. S3 d-f, i, j, m, n, q, r). Thus, PEG could have been taken up by the root tip, and then blocked further water flow into the elongation zone and other upper plant organs.

### Correlation of shoot mass

**Fig. 4:**
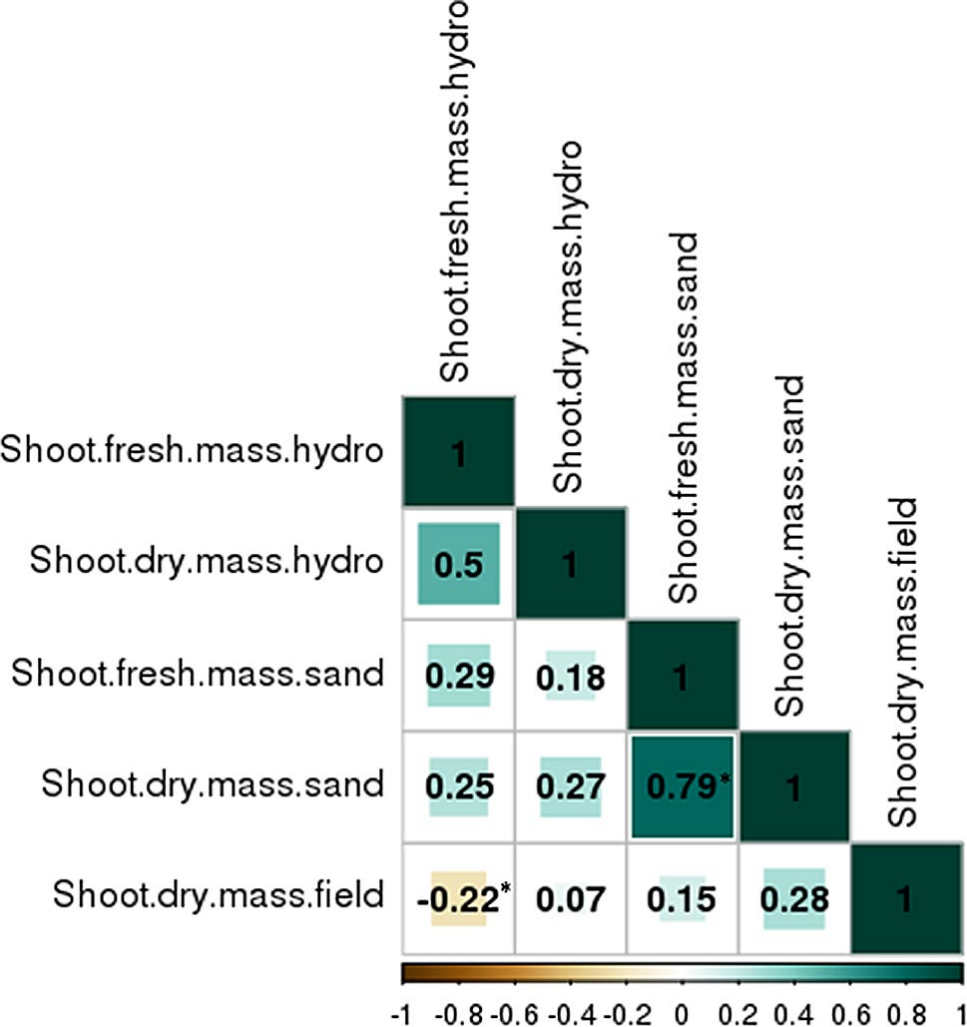
Pearson coefficient of correlation of adjusted means according to Eqs. 2–4 of shoot mass for hydroponic system, sand pots and field trials. Significant correlations (*p* < 0.05) are marked with *, blue = positive correlation, brown = negative correlation. Intensity of the color shows level of correlation

The analyses of root morphology already revealed developmental differences between the two growth conditions. To investigate, which of these methods is more comparable to the drought stress performance under field conditions, we assessed correlations between the shoot mass of the stressed plants grown in a hydroponic system and sand pots, respectively, with the shoot mass of a two-year drought stress experiment under field conditions for the same genotypes. Thereby, the shoot fresh mass for PEG treated plants showed a significant (*p* < 0.05) negative correlation with the shoot dry mass under field conditions (*r* = -0.22) (Fig. 4). In contrast, no correlation was observed between shoot dry mass of PEG treated plants and the shoot dry mass under field conditions. Shoot fresh mass of plants grown in sand showed a slight positive, but not significant correlation with the shoot dry mass field (*r* = 0.15), while the shoot dry mass of plants grown in sand positively correlated with the shoot dry mass field (*r* = 0.28) (Fig. 4). The slightly higher correlation value indicates that the shoot mass of plants grown in sand pots is better comparable to the one from plants grown under field conditions than to the one from plants grown in PEG-enriched hydroponic solution. Possibly, roots grown under field conditions could react to drought stress more similarly to the roots grown in sand-filled pots than to roots stresses with PEG.

## Discussion

Hydroponic systems using PEG as osmotic stressor are a widely used method to investigate the behavior of plants under drought stress. Given the high importance of the roots for water acquisition and uptake, the consequences of PEG treatment on cereal roots are extremely important for interpretation of such studies. In our study, 12.5% (w/v) PEG 6000 was used to simulate drought stress in spring barley from 12 to 33 DAS to investigate several root morphology related parameters. PEG-triggered drought stress experiments applied a concentration of high molecular weight PEG (6000 or 8000) from 5 to 20% (w/v) with an osmotic potential from − 0.27 to -1.09 MPa [35, 36]. The initiation of PEG application for wheat and barley was described between 6 and 31 DAS with a PEG stress duration of 5 to 17 days [37–40]. Thus, our experimental setup was in line with these reported scenarios.

First results of this study showed that all root traits except the root diameter were significantly decreased among all genotypes by PEG (Table 1; Fig. 1). PEG-induced reduction of shoot mass, root length, root surface area, and volume was already described in earlier studies for barley, *Arabidopsis thaliana*, and wheat [13, 40, 41]. In contrast, the root diameter adaptation under drought stress in sand filled pots was the opposite of what was observed by PEG stress (Fig. 1b). This discrepancy could be attributed to the previous described phenomena of PEG caused root thickening as drought stress adaptation mechanism [37, 40]. In particular, Ji et al. [37] pointed out that PEG-treated root tips of wheat were swollen and showed enlarged cortex cells, fragile or broken epidemical cells and a reduced root apical meristem. Moreover, PEG treated root tips contained a higher amount of proline and soluble sugars and less water than untreated root tips. Interestingly, the thickening of the root tip in wheat was observed only between the root apical meristem and the elongation zone, corresponding to the present results (Fig. 2b). In addition, the PEG mediated broken cell membranes and irregular cell structure in this study (Fig. 3, Supplementary Fig. 3) was similarly observed in Chen et al. [42]. In addition, salt stress in a hydroponic system led to an increase in root diameter while exposure to salt stress under field conditions led to a reduction in the size of cortical cells in maize and a reduction of the diameter of epidermal cells and xylem vessels in roots by up to 87% [43–45].

Another explanation for the different root diameter response in sand and PEG could be the observed formation of enlarged vacuoles in the root cells. This special vacuole formation could indicate that PEG was taken up by the roots. The problem of PEG uptake was already pointed out in some studies [16, 46] but was not described for barley roots, yet. For example, PEG 8000 uptake by tomato roots was observed and PEG was even visible in the tomato leaves [47]. Furthermore, PEG uptake rates in maize and beans were reported to be up to one mg/g fresh weight per week [48]. It is possible, therefore, that barley roots also take PEG up and that this uptake causes the significant root thickening in these PEG treated roots. This is an important result with direct consequences for interpretation of other studies. However, it is still not possible to stain or verify intercellular and intracellular PEG in plant cells. PEG itself cannot be visualized by histology or ultrastructure. Nevertheless, it is likely that the changes in the cell organelles were caused by the PEG treatment, as the other conditions for control and PEG were the same and such phenomena have already been detected in other studies, as described above.

**Fig. 5:**
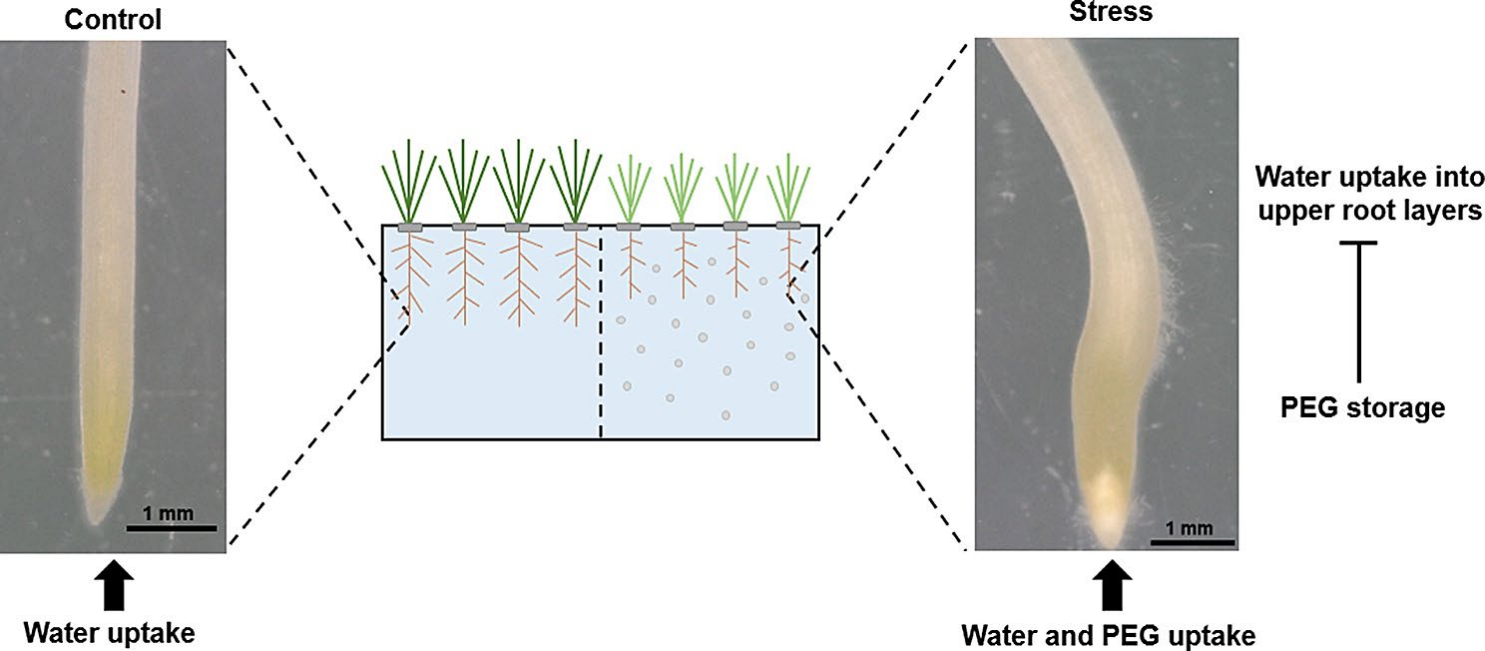
Illustration of water uptake in control plants (left side) and water and PEG uptake in PEG treated plants (right side). Microscopic images of primary roots are from genotype 11 (BCC436). Blue box shows hydroponic system, grey circles PEG and grey square neopren rings. Control plants take up water by e.g. the root tip and transfer it to other plant organs. PEG treated plants take up water and PEG by e.g. the root tip, PEG accumulates in root tip and root tip gets thicker, further water uptake into upper root layers is inhibited by PEG storage

Our observations seem to support the hypothesis that PEG is taken up by the root tip, remains there and thus blocks further water uptake into the upper root layers (Fig. 5). Xiao et al. [49] showed that despite the PEG-mediated increase of the root diameter, the uptake of cadmium into upper root layers was inhibited when wheat was exposed to PEG 6000. Regarding the observed reduction in the diameter of the elongation zone, another study found that there is a direct correlation between PEG treatment with a slower water supply, a thinner root xylem, and reduced symplastic water transport [50]. In addition, a previous study has shown that PEG treated barley roots developed a higher number of suberized cells in the endodermis and the water flow switchds from an apoplastic to a cell-to-cell pathway [51]. Possibly, the shift of the water uptake pathway is caused by PEG uptake of the barley root tips.

The present study presents two different simple phenotyping techniques for root traits determination and the relation to field trials. As mentioned above, differences in several root traits were observed (Fig. 1) through following causes. Firstly, the different kind of drought stressors is likely to play a major role. In case of a PEG treatment, the medium water potential decreases and thereby the absorption of water by plant roots will be disrupted [16]. This approach relies on the simulation of applied osmotic stress by PEG. However, drought stress in sand or soil is based on the interruption of plant watering which decreases soil water content and potential [52]. A second factor could be differences in physical pressure of the surrounding medium, since roots grown in a hydroponic system have lower physical pressure than roots growing in sand or soil. The surrounding pressure of the sand or soil may force the root to longitudinally extend and to laterally contract, whereas without this pressure (i.e. in a hydroponic system), the root system has no incentive to explore for water and nutrition because the roots are already surrounded by water and nutrients. Hence, the root can increase in width without growing in length because of the low physical pressure of the solution.

**Fig. 6:**
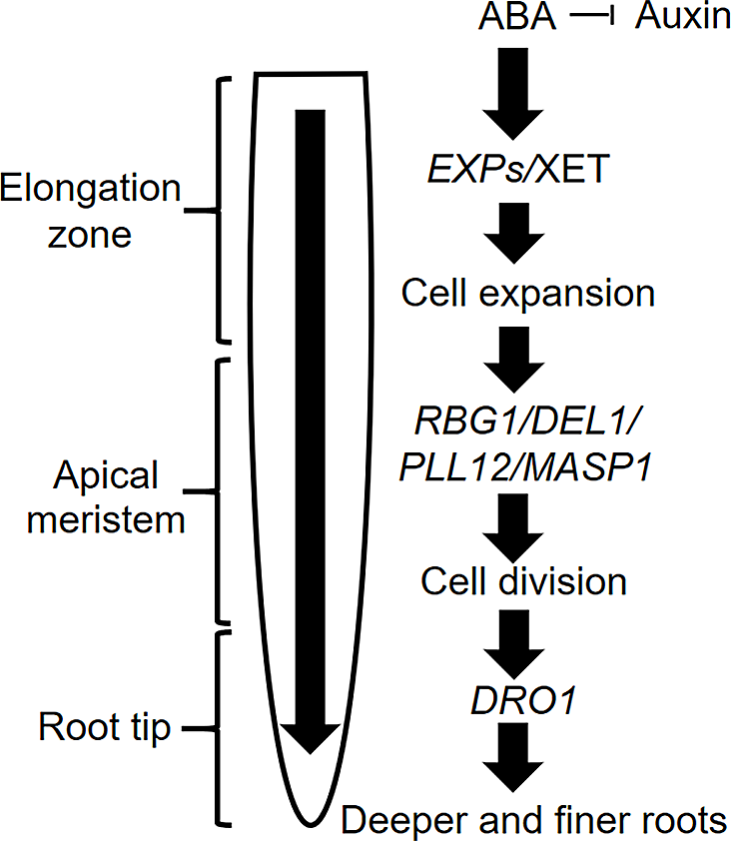
Schematic diagram of root development under drought stress induced in pots or field. *ABA* = abscisic acid, *DEL1* = DP-E2F-like 1, *DRO1* = deeper rooting 1, *EXPs* = expansins, *MASP1* = microtubule-associated stress protein 1, *PLL12* = pectate lyase-like, *RBG1* = rice big grain 1, XET = xyloglucan: xyloglucosyl transferase. Big arrows indicate cascade of root growth, small arrow with horzontal line indicates inhibition

While the reason of root thickening was described before in PEG-related studies, the mechanism of root thinning and deepening was observed in other studies where PEG was not the stressor and illustrated in Fig. 6 [53]. Thereby, plant hormones such as abscisic acid (ABA) and auxin are involved in root architecture alterations by drought stress [54]. Drought stress triggers increased ABA accumulation in the leaf, which is then translocated to the root [55, 56]. Subsequently, ABA in the root inhibits auxin accumulation to promote primary root growth [57], and genes responsible for cell expansion such as expansins (*EXPs*) or proteins like xyloglucan: xyloglucosyl transferase (XET) are induced in the elongation zone [58–60]. Drought stress-related cell division in the apical meristem is then activated by the upregulation of *RBG1*, *DELI1*, *PLLR12*, and *MASP1* [61–63]. Finally, the primary root growth responsible gene *DRO1* located in the root tip triggers further deepening and penetration of the root under drought stress to reach water resources in deeper soil layers [64].

The formation of finer and thinner roots is another important strategy to maintain resources under drought stress in a non-PEG environment [65]. Thinner roots are expected to be more permeable and consequently, facilitate water uptake. In addition, the root length increases as the root diameter decreases, which enables water uptake from deeper soil layers, and this is important during drought stress [9].

Finally, the question arises, which root screening method under controlled conditions is closer to the root development under field conditions. The study of Wasaya et al. [9] compared different root phenotyping techniques and concluded that greenhouse trials with pots filled with soil or sand were closer to the field conditions than experiments with PEG in the laboratory. Interestingly, a comparison of the salt stress tolerance of plants grown in a hydroponic system with those grown in pots or under field conditions showed that grain yield in the field had a higher correlation with the salt tolerance investigated in pots (*r* = 0.79–0.82) than with that studied in the hydroponic system (*r* = -0.22) [66]. Furthermore, the concentration of Na^+^, K^+^ and Cl^−^ in the shoot of plants grown in a hydroponic system were negatively (*r* = -0.23) or not correlated (*r* = 0) with the shoot concentration of plants grown under field conditions whereby it was high significant with the pot trials (*r* = 0.69–0.91) [66]. Our results show a similar tendency regarding the shoot mass (Fig. 4).

Another approach for more field comparable root phenotyping under controlled conditions is the Rhizotron facility. Rhizotrons were developed many years ago [67] and are meanwhile used in an increasing number of automated phenotyping facilities [20, 68, 69]. Nevertheless, access to these facilities is still limited and not always possible. For root morphology analysis, high-resolution X-array tomography (µCT) was described as another method for three-dimensional root scanning beside WinRhizo [70]. However, µCT and WinRhizo scan analysis were compared and µCT method was unable to detect precise root length differences because of segmentation algorithm limitation [71]. Other root traits showed similar trends for both methods.

## Conclusion

In conclusion, for investigations of root traits in barley under controlled drought stress conditions, trials performed in sand-filled pots are a more reliable proxy for field conditions than hydroponic experiments with PEG treatment, because PEG uptake by barley roots can distort results and conclusions in such experiments. Conclusions on root morphology under field conditions based on results from hydroponic systems with PEG must be treated with considerable caution. To relate results and derive conclusions about field conditions, experiments with sand pots appear a considerably better alternative.

## Electronic supplementary material

Below is the link to the electronic supplementary material.

Supplementary Material 1

Supplementary Material 2

Supplementary Material 3

## Data Availability

The data that founded the basis and support the findings of this study are available from the file “Dataset S1.xlsx”.

## Abbreviations

ABA: Abscisic acid
ANOVA: Analysis of variance
BBCH: Federal Biological Research Center for Agriculture and Forestry, Federal Plant Variety Office and Chemical Industry (Biologische Bundesanstalt für Land- und Forstwirtschaft, Bundessortenamt und CHemische Industrie)
Cv: Cultivar
DAS: Days after sowing
LSD: Fisher’s least significance test
PEG: Polyethylene glycol
XET: Xyloglucan-xyloglucosyl
µCT: High-resolution X-array tomography
