# Supplementary material for: PEG treatment is unsuitable to study root related traits as it alters root anatomy in barley (Hordeum vulgare L.)
Source: BMC Plant Biol. 2024 Sep 13;24:856. doi: 10.1186/s12870-024-05529-z (PMC11396634; doi:10.1186/s12870-024-05529-z)
Supplement: Supplementary file 1 — Supplementary Material 1 [file 12870_2024_5529_MOESM1_ESM.pptx]

## Slide 1
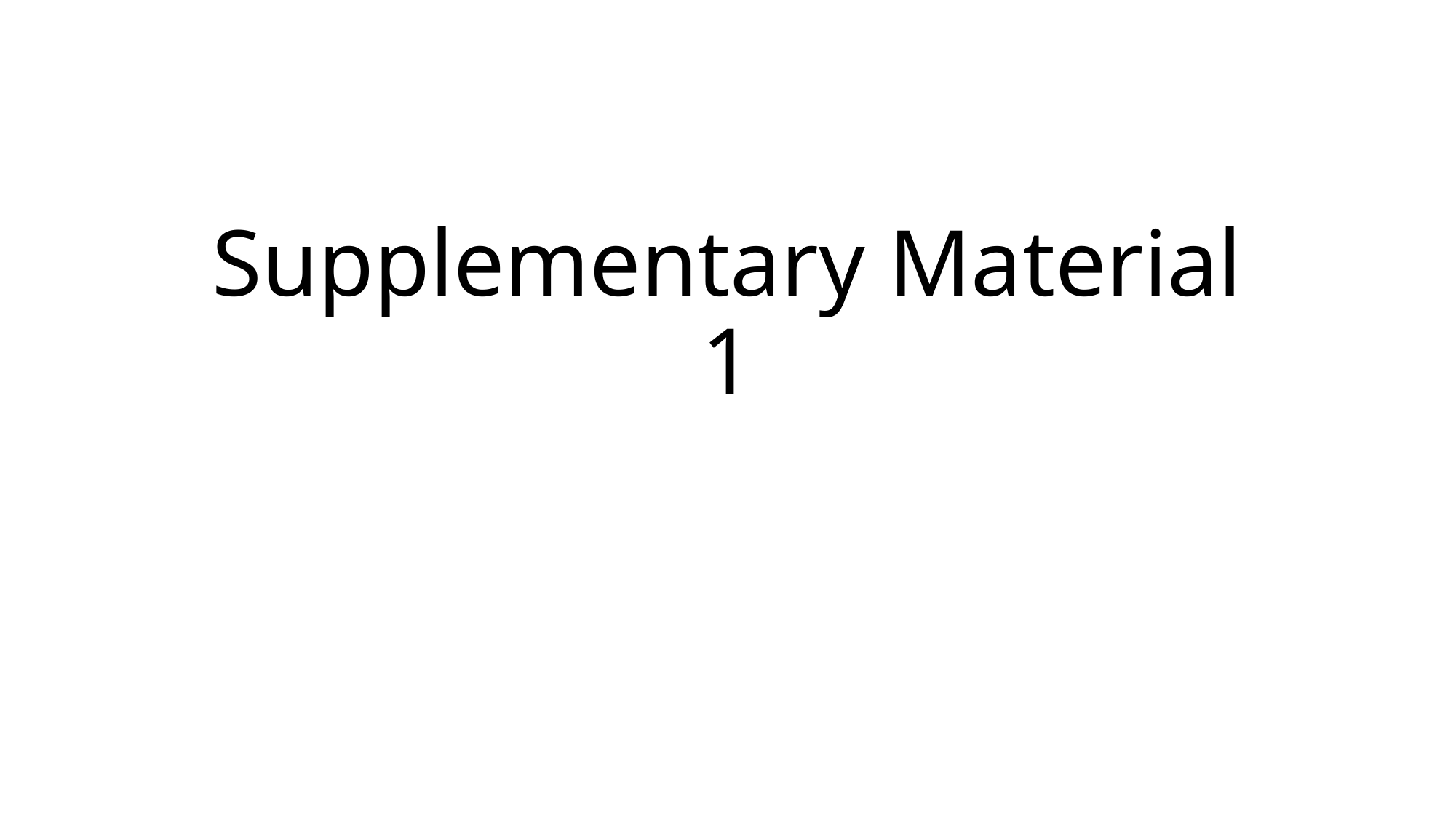

# Supplementary Material 1

## Slide 2
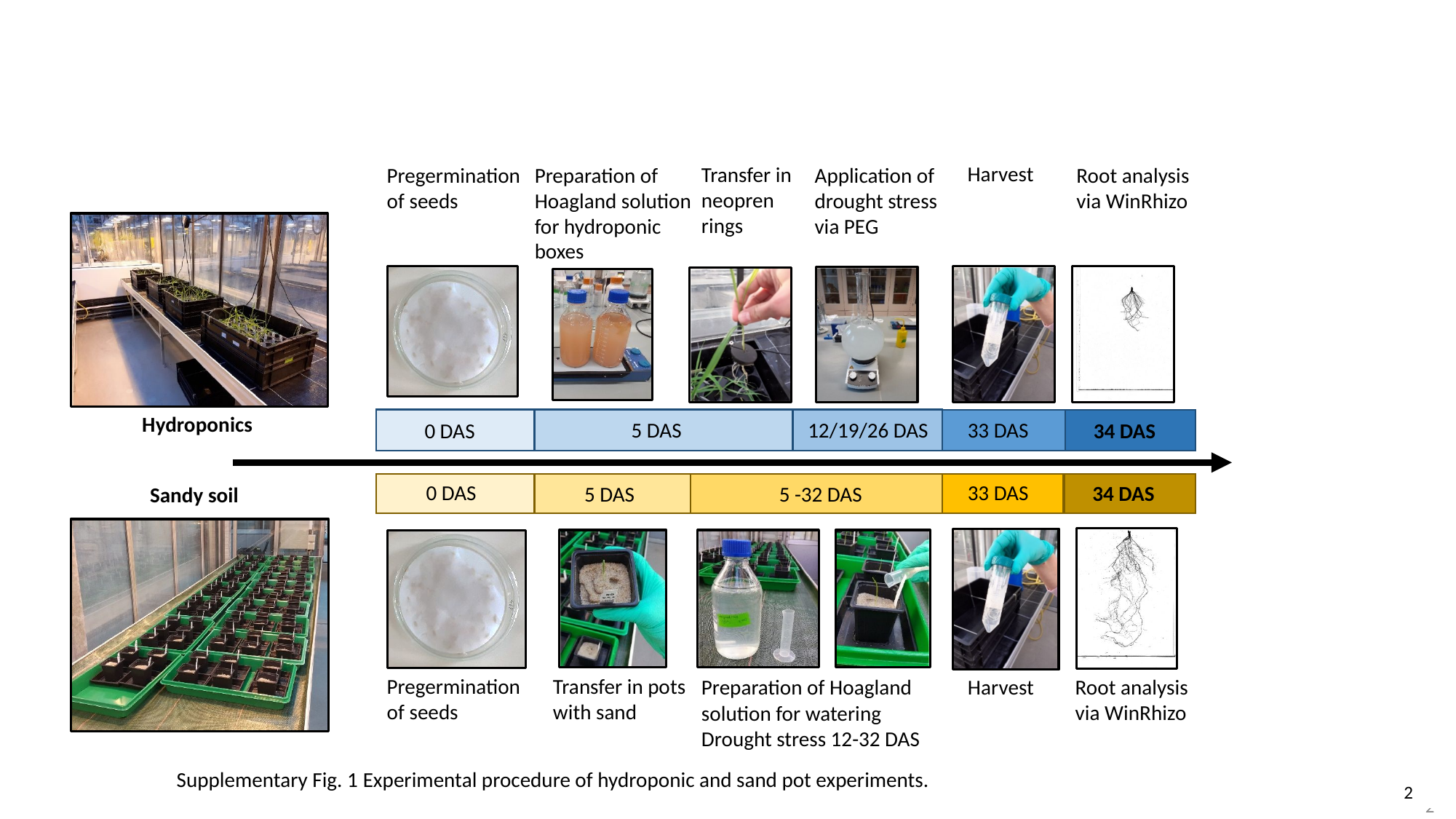

Harvest
Transfer in neopren rings
Pregermination of seeds
Preparation of Hoagland solution for hydroponic boxes
Application of drought stress via PEG
Root analysis
via WinRhizo
Hydroponics
33 DAS
12/19/26 DAS
5 DAS
34 DAS
0 DAS
33 DAS
0 DAS
34 DAS
5 DAS
5 -32 DAS
Sandy soil
Pregermination of seeds
Transfer in pots with sand
Harvest
Root analysis
via WinRhizo
Preparation of Hoagland solution for watering
Drought stress 12-32 DAS
Supplementary Fig. 1 Experimental procedure of hydroponic and sand pot experiments.
2
2

## Slide 3
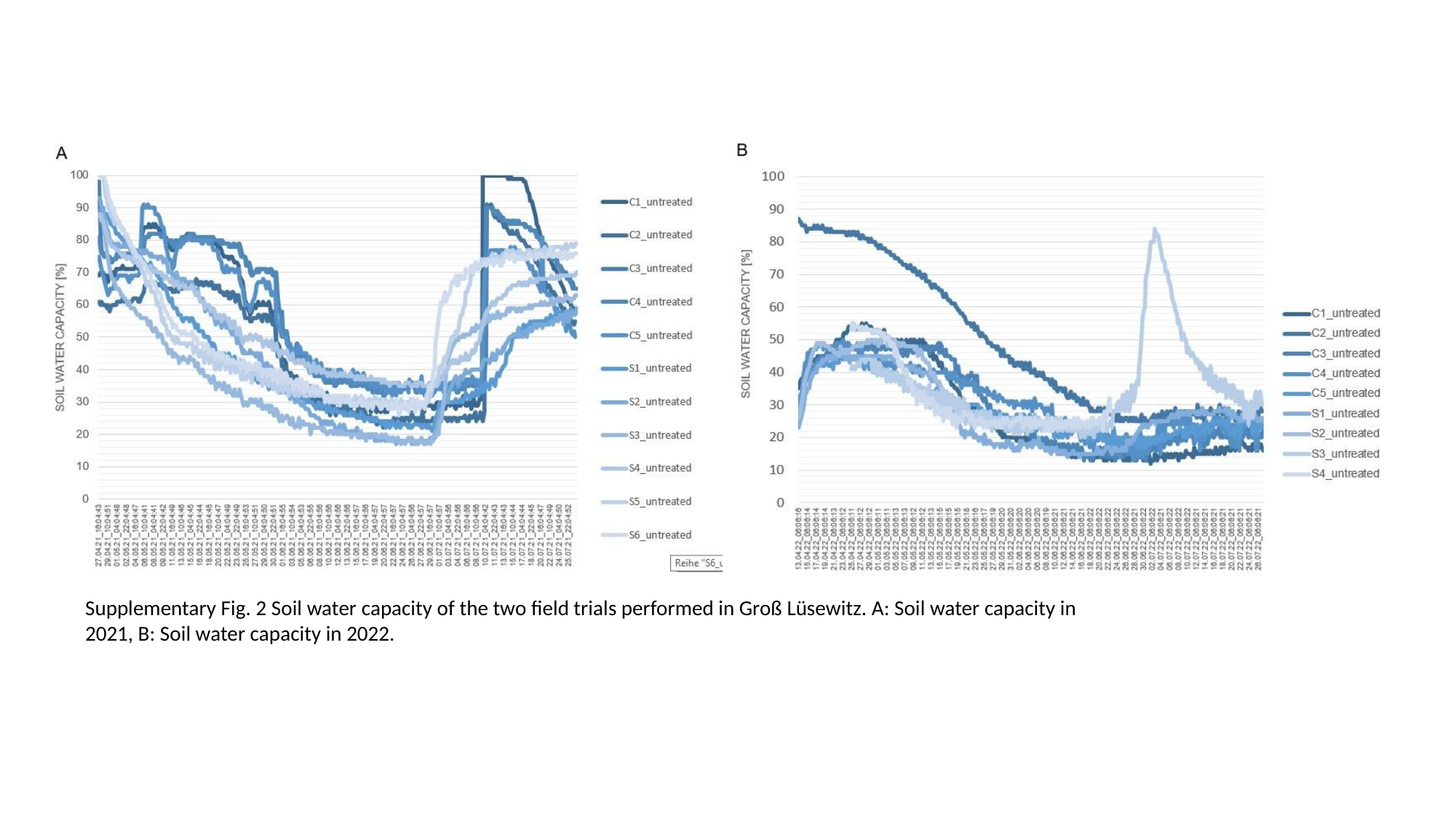

Supplementary Fig. 2 Soil water capacity of the two field trials performed in Groß Lüsewitz. A: Soil water capacity in 2021, B: Soil water capacity in 2022.

## Slide 4
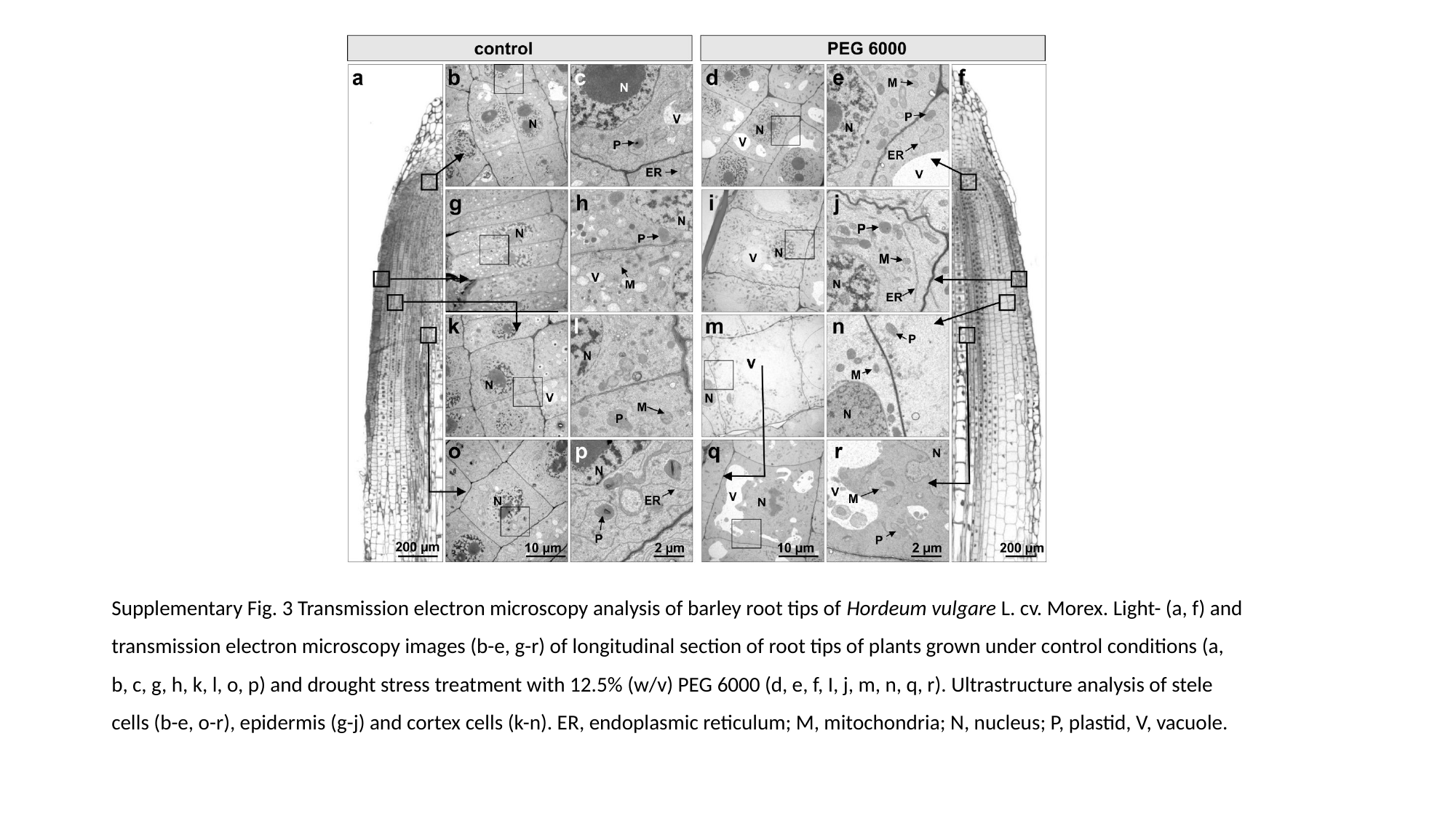

Supplementary Fig. 3 Transmission electron microscopy analysis of barley root tips of Hordeum vulgare L. cv. Morex. Light- (a, f) and transmission electron microscopy images (b-e, g-r) of longitudinal section of root tips of plants grown under control conditions (a, b, c, g, h, k, l, o, p) and drought stress treatment with 12.5% (w/v) PEG 6000 (d, e, f, I, j, m, n, q, r). Ultrastructure analysis of stele cells (b-e, o-r), epidermis (g-j) and cortex cells (k-n). ER, endoplasmic reticulum; M, mitochondria; N, nucleus; P, plastid, V, vacuole.
