# Supplementary material for: PEG treatment is unsuitable to study root related traits as it alters root anatomy in barley (Hordeum vulgare L.)
Source: BMC Plant Biol. 2024 Sep 13;24:856. doi: 10.1186/s12870-024-05529-z (PMC11396634; doi:10.1186/s12870-024-05529-z)
Supplement: Supplementary file 2 — Supplementary Material 2 [file 12870_2024_5529_MOESM2_ESM.docx]

**Supplementary Material 2**

**Supplementary Table 1** Eleven genotypes used for the experiment. NA=not available, UK = United Kingdom, USA = United States of America.

| Genotype No. | Accession number | Country of origin | Ear type |
| --- | --- | --- | --- |
| 1 | JK1 | NA | 6-row |
| 2 | BCC1476 | Uzbekistan | 6-row |
| 3 | BCC1452 | Netherlands | 6-row |
| 4 | BCC812 | Mexico | 2-row |
| 5 | BCC1373 | UK | 2-row |
| 6 | BCC526 | India | 6-row |
| 7 | BCC1455 | Russia | 6-row |
| 8 | Morex | USA | 6-row |
| 9 | Golden Promise | UK | 2-row |
| 10 | BCC1468 | Kazakhstan | 2-row |
| 11 | BCC436 | China | 6-row |

**Supplementary Table 2** List of traits investigated in a hydroponic system and in trials with sand pots.

| **Above ground traits** | **Root traits** |
| --- | --- |
| Root-shoot ratio dry | |
| Root-shoot ratio fresh | |
| Dry matter content [%] | Root dry mass [g] |
| Shoot dry mass [g] | Root fresh mass [g] |
| Shoot fresh mass [g] | Root crossings |
|  | Root diameter [mm] |
|  | Root forks |
|  | Root length [cm] |
|  | Root surface area [mm²] |
|  | Root tips |
|  | Root volume [cm³] |

**Supplementary Table 3** Table of means, minimum, maximum, standard deviation, and covariance among all genotypes for hydroponic experiments. Min = minimum, Max = maximum, STD = standard deviation, CoV = covariance, S/C = Stress/Control.

|  |  | Control | | | | | Stress | | | | |  |
| --- | --- | --- | --- | --- | --- | --- | --- | --- | --- | --- | --- | --- |
| Trait | Unit | Min | Max | Mean | STD | CoV | Min | Max | Mean | STD | CoV | S/C |
| Dry matter content | % | 7.78 | 17.70 | 13.46 | 3.65 | 0.27 | 15.14 | 23.84 | 18.12 | 2.65 | 0.15 | 1.35 |
| Shoot dry mass | g | 0.20 | 0.19 | 0.31 | 0.09 | 0.29 | 0.09 | 0.45 | 0.14 | 0.03 | 0.22 | 0.45 |
| Shoot fresh mass | g | 1.58 | 3.30 | 2.47 | 0.45 | 0.18 | 0.52 | 1.18 | 0.87 | 0.16 | 0.18 | 0.35 |
| Root dry mass | g | 0.02 | 0.03 | 0.02 | 0.00 | 0.21 | 0.02 | 0.02 | 0.02 | 0.00 | 0.12 | 0.77 |
| Root fresh mass | g | 0.42 | 0.79 | 0.65 | 0.12 | 0.19 | 0.36 | 0.53 | 0.45 | 0.06 | 0.13 | 0.70 |
| Root crossings | - | 365.26 | 1064.47 | 589.57 | 209.40 | 0.36 | 61.91 | 163.65 | 106.01 | 29.62 | 0.28 | 0.18 |
| Root diameter | mm | 0.21 | 0.30 | 0.26 | 0.02 | 0.09 | 0.34 | 0.44 | 0.40 | 0.03 | 0.08 | 1.53 |
| Root forks | - | 1704.80 | 3721.49 | 2576.98 | 663.97 | 0.26 | 650.46 | 1102.90 | 843.58 | 153.26 | 0.18 | 0.33 |
| Root length | cm | 342.00 | 655.61 | 463.79 | 96.98 | 0.21 | 124.97 | 192.98 | 162.04 | 19.37 | 0.12 | 0.35 |
| Root-shoot ratio dry | - | 0.07 | 0.14 | 0.11 | 0.02 | 0.18 | 0.12 | 0.21 | 0.15 | 0.03 | 0.17 | 1.45 |
| Root-shoot ratio fresh | - | 0.17 | 0.35 | 0.29 | 0.06 | 0.22 | 0.51 | 1.17 | 0.67 | 0.17 | 0.26 | 2.34 |
| Root surface area | cm² | 26.11 | 46.18 | 38.20 | 6.94 | 0.18 | 16.01 | 24.97 | 20.38 | 2.95 | 0.14 | 0.53 |
| Root tips | - | 1041.93 | 1960.00 | 1475.76 | 315.96 | 0.21 | 402.95 | 576.95 | 505.07 | 56.53 | 0.11 | 0.34 |
| Root volume | cm³ | 0.16 | 0.32 | 0.26 | 0.05 | 0.21 | 0.16 | 0.27 | 0.21 | 0.04 | 0.20 | 0.81 |

**Supplementary Table 4** Table of means, minimum, maximum, standard deviation, and covariance among all genotypes for sand pot experiments.

|  |  | Control | | | | | Stress | | | | |  |
| --- | --- | --- | --- | --- | --- | --- | --- | --- | --- | --- | --- | --- |
| Trait | Unit | Min | Max | Mean | STD | CoV | Min | Max | Mean | STD | CoV | S/C |
| Dry matter content | % | 9.6 | 10.92 | 10.25 | 0.4 | 0.04 | 11.48 | 15.33 | 13.13 | 1.29 | 0.10 | 1.28 |
| Shoot dry mass | g | 0.08 | 0.16 | 0.11 | 0.02 | 0.16 | 0.06 | 0.08 | 0.06 | 0.01 | 0.11 | 0.57 |
| Shoot fresh mass | g | 0.87 | 1.60 | 1.15 | 0.19 | 0.17 | 0.42 | 0.6 | 0.52 | 0.06 | 0.11 | 0.45 |
| Root dry mass | g | 0.03 | 0.05 | 0.04 | 0.00 | 0.13 | 0.03 | 0.04 | 0.03 | 0.00 | 0.08 | 0.83 |
| Root fresh mass | g | 0.71 | 1.22 | 0.98 | 0.15 | 0.15 | 0.37 | 0.49 | 0.42 | 0.04 | 0.1 | 0.43 |
| Root crossings | - | 138.7 | 404.78 | 269.96 | 82.59 | 0.31 | 102.59 | 300.12 | 204.73 | 49.8 | 0.24 | 0.76 |
| Root diameter | mm | 0.35 | 0.50 | 0.41 | 0.04 | 0.09 | 0.32 | 0.42 | 0.36 | 0.03 | 0.07 | 0.89 |
| Root forks | - | 1923.21 | 4023.87 | 2732.1 | 629.77 | 0.23 | 1248.77 | 2100.63 | 1714.08 | 260.83 | 0.15 | 0.63 |
| Root length | cm | 260.59 | 425.98 | 323.35 | 53.67 | 0.17 | 176.79 | 287.2 | 245.24 | 31.32 | 0.13 | 0.76 |
| Root-shoot ratio dry | - | 0.29 | 0.41 | 0.34 | 0.03 | 0.1 | 0.40 | 0.58 | 0.50 | 0.05 | 0.10 | 1.47 |
| Root-shoot ratio fresh | - | 0.73 | 0.98 | 0.78 | 0.05 | 0.06 | 0.67 | 1.1 | 0.92 | 0.07 | 0.07 | 1.18 |
| Root surface area | cm² | 33.09 | 60.67 | 42.78 | 6.98 | 0.16 | 23.4 | 32.23 | 27.84 | 2.7 | 0.10 | 0.65 |
| Root tips | - | 577.29 | 1108.14 | 822.08 | 208.76 | 0.25 | 350.01 | 597.08 | 470.03 | 84.84 | 0.18 | 0.57 |
| Root volume | cm³ | 0.34 | 0.68 | 0.46 | 0.09 | 0.2 | 0.22 | 0.31 | 0.26 | 0.03 | 0.1 | 0.56 |
